# Monoclonal Antibodies (mAbs) and Proteins: The Biologic Drugs Approved by the Food and Drug Administration (FDA) in 2024

**DOI:** 10.3390/biomedicines13081962

**Published:** 2025-08-12

**Authors:** Alexander C. Martins, Mariana Y. Oshiro, Beatriz N. Schiavon, Glaucia A. de Jesus, Beatriz G. de la Torre, Fernando Albericio

**Affiliations:** 1 Medical Communication Department, IQVIA RDS, São Paulo 04719-002, Brazil; 2 Escola de Ciências da Saúde, UAM—Universidade Anhembi-Morumbi, São Paulo 03101-001, Brazil; mariyukie@hotmail.com; 3 Cancer Center, A. C. Camargo (Antônio Cândido de Camargo), São Paulo 01508-010, Brazil; drabeatriz.nschiavon@gmail.com; 4 Escola de Medicina, Universidade Nove de Julho, São Paulo 07013-110, Brazil; g.angelina@uni9.edu.br; 5 School of Laboratory Medicine and Medical Sciences, College of Health Sciences, University of KwaZulu-Natal, Durban 4001, South Africa; garciadelatorreb@ukzn.ac.za; 6 School of Chemistry and Physics, University of KwaZulu-Natal, Durban 4001, South Africa; 7 Department of Organic Chemistry, University of Barcelona, 08028 Barcelona, Spain

**Keywords:** Food and Drug Administration, FDA, monoclonal antibody, biologics, cancer, first approval, Alzheimer’s disease

## Abstract

Advances in drug development continue to play a critical role in addressing diseases, including those with unmet medical needs. In 2024, the FDA approved 50 novel drugs, 16 of which were biologics. For context, during the first half of 2024 alone, the agency approved six biologics. By mid-2025, six additional biologics have received the green light, indicating that the pace of approvals of this class of drugs this year may be on par with 2024. This paper analyzes all biologics that received FDA authorization in 2024, examining their mechanisms, clinical trials, and expedited review pathways. Key approvals included the highest number of monoclonal antibodies (mAbs) since 2015 (13 mAbs, 6 indicated for oncology), while no antibody–drug conjugates were authorized—continuing with the trend in 2023. In addition, a new chimeric mAb has been approved since the last chimeric mAb approved in 2022, and a new mAb for Alzheimer’s disease. Nine biologics are first-in-class therapies, while ten received Orphan Drug Designation. The biologics considered herein fall into the categories of mAbs and proteins.

## 1. Introduction

Drug authorizations by the US Food and Drug Administration (FDA) totaled 50 in 2024, matching the five-year average (49) and ten-year average (43), thereby indicating stability in approval rates. Although the number of New Chemical Entities (NCEs) exceeded that of biologics (170 vs. 75 over the last five years and 292 vs. 139 over the last 10 years), biologics still represented approximately one-third of all approved drugs. In this regard, in 2024, biologics made up 32% of drug approvals (16 out of 50), consistent with the 10-year trend (139 out of 431 approvals). Importantly, 68% (34) of the total number of drugs to receive authorization in 2024 were approved in the United States of America (USA) before any other country [1]. Table 1 illustrates this trend.

**Figure 1:**
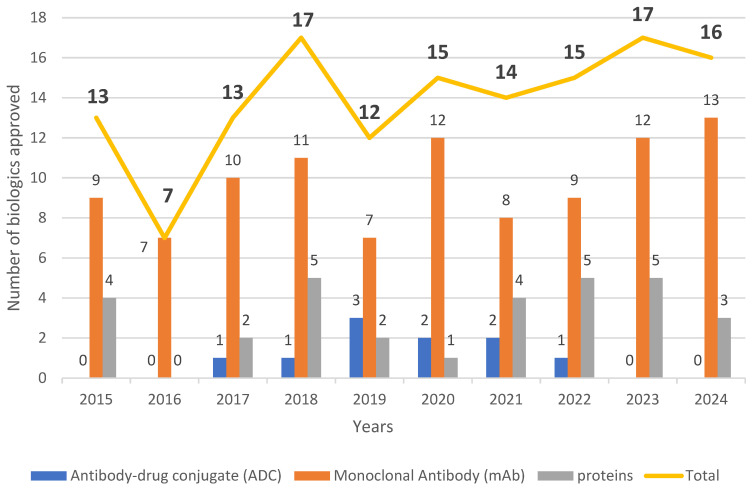
Biologics approved by the FDA from 2015 to 2024. This figure includes publicly available data from FDA databases.

**Figure 2:**
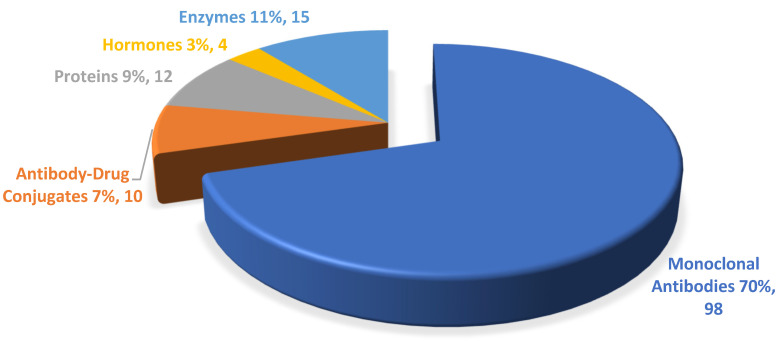
Percentage of new biologics approved by the FDA from 2015 to 2024. This figure includes publicly available data from FDA databases.

In 2024, 13 monoclonal antibodies (mAbs) and three proteins received the green light. Among the mAbs, one chimeric and three bispecific mAbs were approved. No antibody–drug conjugate (ADC) or hormones received authorization. Figure 1 and Figure 2 show the trend in these categories over the last 10 years.

## 2. Analysis

Table 2 shows all the biologics approved by the FDA in 2024.

As in previous years, cancer was the disease most targeted by the biologics approved in 2024; however, unlike previous years, it was the year with the fewest biologics targeting autoimmune conditions and/or diseases somehow related to autoimmune responses (but not primarily being an autoimmune condition, such as chronic graft-versus-host disease). First-in-class drugs—characterized by a unique mechanism of action, a novel target in a disease, or a distinct pharmacologic effect—accounted for 24 out of the 50 drugs to receive the green light in 2024. Amongst the 16 biologics approved, the following nine were first–in-class: zanidatamab; sotatercept; zolbetuximab; axatilimab; nemolizumab; tarlatamab; marstacimab; zenocutuzumab; and nogapendekin alfa inbakicept [1]. Three hematologic biologics, namely concizumab, marstacimab, and crovalimab, also received authorization in 2024.

This article explores in detail several key aspects of the biologics approved in 2024, including those that received Orphan Drug Designation (ODD) and those that used FDA expedited programs. It also covers first-in-class biologics as defined by the FDA and the Center for Drug Evaluation and Research (CDER), their mechanisms of action, and the outcomes of the major clinical trials for the most important approvals. Furthermore, the article analyzes drug approvals year by year, examines the correlation with ODD approvals, and highlights key findings of 2024.

Table 3 provides a quantitative analysis of FDA-granted Orphan Drug Designations.

Notably, 52% (26) of the 50 drugs approved in 2024 are indicated to treat rare or orphan diseases [1].

In our previous article, we also discussed the FDA expedited drug development programs, and 2024 was no different. In this regard, 26 of the 50 drug approvals received ODD, and 33 used one or more expedited programs [1]. Of the biologics, concizumab received ODD as well as the Priority Review Designation, which provides additional resources for the evaluation process and shortens the review process to two months, compared to the standard six months under the traditional system [28,29].

Zenocutuzumab and zanidatamab received Priority Review, Breakthrough Therapy Designation, and ODD [24,25,30]. Axatilimab also received ODD, Priority Review, and Fast Track Designation for its therapeutic indication [20]. Tarlatamab received ODD, Breakthrough Therapy Designation, and Accelerated Approval, as well as Priority Review and participation in the Project Orbis, which grants collaboration with regulatory agencies all over the world, thereby promoting faster access to new clinical therapies for patients [16]. Sotatercept received ODD and Breakthrough Therapy Designation [30]. The remaining biologics to be granted differentiated regulatory advantages, namely marstacimab, received the ODD and first-in-class designation; zolbetuximab received ODD and first-in-class designation, and it also benefited from the Fast Track and Priority Review, crovalimab received only the ODD designation; and tislelizumab received only the ODD designation. Regarding the biologics that received ODD for certain therapeutic indications, this article aims to clarify the distinction between full ODD approval for a specific indication and ODD status without subsequent approval. Notably, some drugs may be granted ODD for certain indications but are never ultimately authorized for those indications and subsequent clinical use. The key difference between the two categories lies in the inclusion of the indications in the drug’s prescribing information: only indications that achieve full ODD approval from the FDA are listed in the official labeling, whereas indications are omitted for those granted only ODD without subsequent approval. In total, 10 biologics received ODD in 2024.

In 2024, no ADCs received authorization, continuing the trend observed in 2023. Neither were any hormones approved. However, another biologic for Alzheimer’s disease (AD) did receive the green light, confirming the commitment shown by pharmaceutical companies in previous years to address this condition. Furthermore, 2024 witnessed the approval of more hematologic biologics, as well as three bispecific mAbs and a new chimeric mAb.

Table 4 provides more details regarding the approvals of biologics and ODD in 2024, including both of the following: (1) drugs and their approved therapeutic indications (i.e., those that received ODD and subsequent approval for the orphan indication and subsequent clinical use) and (2) drugs that received ODD but were not ultimately approved for the orphan indication (i.e., the indications are not listed in the prescribing information).

Fundamentally, the indication approved and found in the prescribing information of lebrikizumab is a non-ODD-eligible drug (moderate to severe atopic dermatitis not satisfactorily controlled with topical medicines). It is important to note that biologics can receive ODD for a specific target disease but may not necessarily receive FDA approval for that orphan indication. Obtaining both the designation and subsequent approval completes the full regulatory process. The therapeutic indication for lebrikizumab that was not approved for ODD is idiopathic pulmonary fibrosis (IPF). Through the NCT01872689 identifier, it is possible to find a completed Phase II study of lebrikizumab in IPF, but the results published from that study are related to additional variants in the genome related to IPF risk. A Phase III trial scheduled in the near future may provide new information on this topic.

## 3. Biologics in 2024

### 3.1. Biologics for Cancer

#### 3.1.1. Tevimbra^TM^ (Tislelizumab)

The incidence of and mortality from esophageal squamous cell carcinoma (ESCC) have been rising annually, with over 600,000 new cases and over 540,000 deaths reported in 2020, respectively. Therefore, advancements in treatment and research into new biomarkers for this type of cancer, are urgently needed and highly welcomed. Although the incidence of ESCC is falling in some countries (e.g., USA, UK, and China) due to various factors, it continues to be the leading cause of cancer-related death in others (Bangladesh and Malawi) [31,32].

The most recent approval by the FDA, Tevimbra^TM^ (tislelizumab/also found in the literature as BGB-A317), a humanized IgG4 kappa mAb indicated for unresectable or metastatic ESCC, releases the immune response pathway by decreasing the suppression of T-cell proliferation and cytokine production. It achieves this by binding to PD-1 receptors found on T cells, thereby inhibiting their interaction with the ligands PD-L1 and PD-L2 [13,31,33].

Unlike other anti-PD1 antibodies, such as pembrolizumab and nivolumab, tislelizumab has a lower affinity for FcγRI (Fc gamma receptor I) expressed in type 2 macrophages in particular conditions, such as certain tumors and inflammatory processes, and thus does not mediate the crosslink between FcγRI and PD-1 [34]. These characteristics confer this new mAb a higher anti-tumor efficacy [13]. Tislelizumab also has a higher association rate (*K*a) with PD-1, binding to PD-1 at a rate of 575,000 per molar per second, than nivolumab or pembrolizumab, but a slower dissociation rate (Kd) from PD-1, around 100-fold and 50-fold slower compared to the other two mAbs [35]. These features provide unique traits to tislelizumab, such as kinetics, binding affinity, and blocking activity. Studies in mouse models demonstrated that these features confer this biologic a longer half-life, around 30-to-80-fold longer than that of pembrolizumab and nivolumab [36]. However, in the latest updated prescribing information for these biologics (already on the market), the half-life shows little variation: half-life of 24 days for tislelizumab [33], 25 days for nivolumab [37], and 22 days for pembrolizumab [38].

In a Phase III clinical trial (RATIONALE-302), 197 deaths were finally registered in the tislelizumab arm and 213 deaths in the chemotherapy group (investigator’s choice of paclitaxel, docetaxel, or irinotecan), with a higher overall survival (OS) for patients treated with tislelizumab, with a median of 8.6 months vs. 6.3 months for chemotherapy, and a 12-month OS of 37.4% vs. 23.7% for the chemotherapy group [39].

#### 3.1.2. Anktiva^TM^ (Nogapendekin Alfa Inbakicept)

Nogapendekin alfa inbakicept (also known as N803) is a recombinant interleukin-15 (IL-15) receptor superagonist. It is a soluble complex consisting of nogapendekin alfa (a human IL-15N72D variant, presenting 114 amino acids) bound to inbakicept [a dimeric human IL-15Rα sushi domain (with 65 amino acids)/human IgG1 Fc fusion protein (232 amino acids)]. It is indicated with Bacillus Calmette–Guérin (BCG) for the treatment of adult patients with BCG-unresponsive non-muscle invasive bladder cancer (NMIBC) with carcinoma in situ (CIS), with or without papillary tumors [40]. This novel cancer treatment strongly promotes the activation, proliferation, and cytolytic function of Natural Killer (NK) cells and CD8+ T cells, inducing pleiotropic immune effect supportive of tumor suppression when administered as a monotherapy and in combination with other agents [41].

N803 was approved in the USA in April 2024 for the treatment of adult patients with NMIBC with CIS, with or without papillary tumors [42]. However, it has also been used to treat other kinds of solid cancers, including resectable head and neck squamous cell carcinoma not associated with human papillomavirus infection [43], HIV infection [44], and more recently, a case report of advanced metastatic pancreatic cancer [45].

In 2018, 20 healthy volunteers were administered N803 subcutaneously (s.c.) at 10 µg/kg on the first day of study period 1 and were monitored for 8 additional days. After at least 6 days, study period 2 was initiated, whereby 14 of these subjects received an additional dose of N803 (s.c.) at 20 µg/kg on the first day. N803 demonstrated a half-life of ~20 h, which is over 20-fold that of rhIL-15 (Recombinant Human Interleukin-15). N803 was well tolerated, and no serious adverse events (AEs) or grade ≥ 3 AEs were observed [41].

According to the manufacturer, the efficacy of N803 was evaluated in QUILT-3.032 (NCT03022825), a single-arm, multicenter trial involving 77 adult patients with BCG-unresponsive, high-risk, NMIBC with CIS, with or without Ta/T1 papillary disease, after transurethral resection. The median age of patients was 73 years (range: 50–91 years) and 86% were male. Baseline high-risk NMIBC disease status was 43% refractory and 57% relapsed. The median number of prior BCG doses received was 12 (range: 8–45 doses); 13% received prior partial-dose BCG. Complete Response Rate was around 95% CI [40].

Further studies with a larger number of individuals are needed to ensure the long-term safety, quality, and efficacy of this medication.

#### 3.1.3. Imdelltra^TM^ (Tarlatamab)

Tarlatamab is a first-in-class, half-life extended bispecific antibody delta-like ligand 3 (DLL3)-directed CD3 T cell engager indicated for the treatment of adult patients presenting advanced stage small cell lung cancer (ES-SCLC) with disease progression during or after platinum-based chemotherapy. This drug was approved in 2024 under accelerated approval based on overall response rate and duration of response [46,47].

SCLC accounts for approximately 15% of all lung cancers. Treatment options are very limited for recurrent or refractory disease, and those available are associated with significant high rates of toxicity and collateral effects. DLL3 is an inhibitory Notch ligand that is highly expressed in SCLC and other neuroendocrine tumors but minimally expressed in healthy tissues, which is why it has been explored as a potential therapeutic target in SCLC [48].

To date, the only trial addressing tarlatamab (NCT03319940) has given rise to a number of publications. The most recent study reported an Objective Response Rate (ORR) of 25%, a median duration of response (mDOR) of around 11.2 months, and a median overall survival (mOS) of about 17.5 months in cohorts treated with ≥10 mg of tarlatamab once every two weeks, once every three weeks, or once on day 1 and once on day 8 of a 21-day cycle. For 17 patients receiving only 10 mg of tarlatamab once every two weeks, the ORR was 35.3%, the mDOR was 14.9 months, the mOS was 20.3 months, and 29.4% of the patients treated had sustained disease control over time on treatment ≥ 52 weeks [49]. Furthermore, according to the same study, no new safety concerns were identified.

Another study from the same trial reported an ORR of around 23.4%, including 2 complete and 23 partial responses. The mDOR was around 12.3 months, and 51.4% of patients treated showed disease control. The median progression-free survival and OS were around 3.7 months and 13.2 months, respectively. This study concluded that tarlatamab demonstrated manageable safety with encouraging response durability in patients presenting with heavily pretreated SCLC who had not achieved a significant response [50].

A Phase II, open-label, randomized, multicenter study of tarlatamab dosing regimens in subjects with SCLC (DeLLphi-309) is recruiting patients for a new evaluation of this drug (NCT06745323). The results are expected in the coming years [51].

#### 3.1.4. Vyloy^TM^ (Zolbetuximab)

Esophageal and gastric cancers are among the most common types of cancer worldwide. In 2018, there were about 1.6 million new cases and 1.3 million deaths from these diseases. The number of cases of gastroesophageal junction (GEJ) cancer has been rising, especially in the USA, where one study showed a 2.5-fold increase in the past 20 years [52].

Zolbetuximab was approved by the FDA on October 2024. This drug is a mAb, a first-line treatment for patients with HER2-(negative). Targeting cloudin protein 18 isoform 2 (CLDN18.2), a tight-junction protein overexpressed in gastric and gastroesophageal junction (GEJ) tumors whose function is to maintain tight-junction integrity, but when overexpressed, may promote cancer cell survival and invasion, it is administered along with chemotherapy, which includes fluoropyrimidine and platinum-based drugs [53].

In the SPOTLIGHT trial (NCT03504397), 565 patients randomly received either zolbetuximab plus mFOLFOX6 or placebo plus mFOLFOX6. The results showed that patients treated with zolbetuximab achieved a median progression-free survival of 10.61 months (95% CI: 8.90–12.48) vs. 8.67 months (95% CI: 8.21–10.28) in the placebo group. Zolbetuximab treatment significantly reduced the risk of disease progression or death compared to placebo. Treatment-related deaths were reported in 5 patients (2%) receiving the drug, compared to 4 patients (1%) in the placebo group. This Phase III trial (SPOTLIGHT) showed significantly prolonged progression-free survival and OS of patients with CLDN18.2+ (positive), HER2-(negative) gastric, or GEJ cancer treated with zolbetuximab plus mFOLFOX6, compared to placebo plus mFOLFOX6 [54].

In the GLOW trial, 507 patients randomly received either zolbetuximab with CAPOX chemotherapy or placebo with CAPOX chemotherapy. In this Phase III trial [55], zolbetuximab met the primary endpoint by significantly improving median progression-free survival to 8.21 months, compared to 6.80 months in the placebo group [56].

For adult patients with metastatic gastric cancer, GEJ cancer, or esophageal adenocarcinoma, several drugs with mechanisms of action that differ from that of zolbetumximab are available. Among them, pembrolizumab and nivolumab are considered first-line treatment for tumors that express PD-L1. In patients with HER2+ (positive) tumors, trastuzumab deruxtecan has emerged as a promising second-line treatment option after trastuzumab stops being effective [57].

#### 3.1.5. Ziihera^TM^ (Zanidatamab)

Zanidatamab is a cytolytic bispecific IgG1-like HER2-directed mAb indicated for adult patients who have previously treated unresectable or metastatic HER2+ (IHC 3+) biliary tract cancer (BTC), as detected by an FDA-approved test [58]. More specifically, zanidatamab (also found as ZW25 in the literature) is a humanized, biparatopic, bispecific IgGκ assembled from half-antibodies, and it targets two non-overlapping epitopes of HER2 [59].

This is the first antibody to bind two distinct sites on HER2 in HER2-expressing solid tumors [60], and it inhibits tumor growth and cell death in vitro and in vivo. It acts by complement-dependent cytotoxicity (CDC), antibody-dependent cellular cytotoxicity (ADCC), and antibody-dependent cellular phagocytosis (ADCP) activity. Zanidatamab is a new molecular entity that has not been marketed previously [61]. This drug was approved in November 2024, and there are several ongoing clinical trials in the FDA database, nine of which are recruiting for testing.

Despite being a new molecule, zanidatamab has already been used to treat different HER2+ (positive) tumor types, including locally advanced or metastatic BTC [62], extrahepatic cholangiocarcinoma, and gallbladder cancer [63], gastroesophageal adenocarcinoma [64], and advanced endometrial carcinoma and carcinosarcoma [65]. Zanidatamab brings new hope for the treatment of HER2-overexpressed tumors for which conventional chemotherapy has been unsuccessful.

According to [59], the approval of this drug was based, in part, on the results of the single-arm, Phase IIb trial (HERIZON-BTC-01; NCT04466891) in patients with HER2-amplified, unresectable, locally advanced, or metastatic BTC with disease progression on previous gemcitabine-based therapy. Patients were assigned to cohorts based on the HER2 immunohistochemistry (IHC) score: Cohort 1 (IHC 2+ or 3+; HER2+) and Cohort 2 (IHC 0 or 1+). In addition to BTC, zanidatamab is also being evaluated in Phase III studies as a treatment for HER2+ breast cancer (NCT06435429) and gastric cancer (NCT05152147).

As BTCs (cholangiocarcinomas and gallbladder cancers) are increasing in incidence and present a poor prognosis, most studies using zanidatamab address these types of tumors. Several targeted therapies have been developed, and this antibody has shown encouraging response rates [66]. It is a promising drug, and the results of the trials are eagerly anticipated. However, more studies are necessary to demonstrate the long-term effectiveness of the medication while controlling eventual collateral effects.

#### 3.1.6. Bizengri^TM^ (Zenocutuzumab)

Zenocutuzumab is an antibody indicated to treat adult patients with advanced unresectable or metastatic non-small cell lung cancer (NSCLC) harboring a neuregulin 1 (NRG1) gene fusion with disease progression on or after prior systemic therapy [67]. More specifically, it is an IgG1 bispecific human epidermal growth factor receptor HER2- and HER3-directed antibody for the treatment of solid tumors with NRG1 gene fusions, and it was first approved on 4 December 2024 in the USA [68]. NRG1 fusions have been detected with a wide range of fusion partners, both across and within various types of cancer [69], such as pancreatic cancer, another indication for this biologic, which is refractory to most forms of conventional treatment, demonstrating poor survival outcomes [70].

NSCLC is one of the most frequent types of cancer and is responsible for most cancer-related deaths worldwide [71]. NSCLC is a heterogeneous category of malignancies that includes several types of cancer, with lung adenocarcinoma being the most common histologic subtype (∼50%) [72] and associated with very poor survival outcomes. Due to poor survival and limited response to current treatments, the FDA accelerated the approval of zenocutuzumab [69].

There is a Phase I/II, open-label, multicenter, multi-national, dose escalation study in progress [73] to assess the safety, tolerability, immunogenicity, and anti-tumor activity of zenocutuzumab. The main goal of this trial is to determine the percentage of patients whose tumors decrease in size by 30% or more. Two other studies have already ended (NCT03321981 and NCT05588609); however, no results have been published to date. There is little information about this treatment in the literature. More studies are necessary to elucidate the effectiveness of this drug.

#### 3.1.7. Unloxcyt^TM^ (Cosibelimab)

Cosibelimab is a human unmodified immunoglobulin G1 IgG1 mAb [74] that belongs to a group of drugs that bind to the programmed death receptor-1 (PD-1) or PD-ligand 1 (PD-L1), blocking the PD-1/PD-L1 pathway and lifting the inhibition of the immune response, potentially disrupting peripheral tolerance and inducing immune-mediated adverse effects [75]. First approved in December 2024, it was developed by Checkpoint Therapeutics for the treatment of advanced cancers, including metastatic or locally advanced cutaneous squamous cell carcinoma (mCSCC or laCSCC) [76]

As this antibody lifts the inhibition of the immune response, leading to immune-mediated adverse effects, this treatment may result in a range of serious or fatal immune-mediated reactions. Although immune-mediated adverse reactions usually manifest during treatment with PD-1/PD-L1 blocking antibodies, they can also occur after discontinuation of this treatment [77].

Cutaneous squamous cell carcinoma (CSCC) is the second-most common skin cancer. Although patients with this disease often present a favorable prognosis and a high likelihood of long-term survival after surgical excision, those with mCSCC or laCSCC who are not eligible for curative surgery or radiation have limited treatment options. While current immunotherapies targeting PD-1 have proven effective in mCSCC, some patients experience inadequate responses or severe immune-related adverse events, including fatigue, rash, anemia, diarrhea, and others, and there remains an unmet need to improve outcomes, tolerability, and safety [74].

The efficacy of cosibelimab was evaluated in a multicenter, multicohort, open-label trial, CK-301-101 (NCT03212404), including 109 patients with mCSCC or laCSCC who were not candidates for curative surgery or curative radiation [26]. No further results have been published; the study is still in progress and pending completion.

According to the study [78], the Phase II clinical trial (NCT03212404) highlighted the strong efficacy of cosibelimab in the treatment of advanced CSCC. This trial showed an ORR of 47.5%, with 7% of patients achieving complete responses and 40.5% achieving partial responses, indicating that approximately half the patients experienced significant tumor reduction or elimination. Progress-free survival was reported at 12.9 months, suggesting that patients could maintain disease control for over a year on average. The median time to response to treatment was approximately 11.3 months, highlighting the long duration of therapeutic effects, once this treatment achieved the expected response. Early OS data showed a median of 18.4 months, suggesting an increase in life expectancy for these patients.

The results of the NCT03212404 trial showed the efficacy of cosibelimab regardless of PD-L1 expression levels, thereby reinforcing its potential as a therapeutic option for these aggressive skin malignancies [78]. The study [79] also reported significant results regarding the response rate of patients with mCSCC or laCSCC who are not candidates for surgery or radiotherapy.

Although cosibelimab is a promising treatment with a high response rate, the side effects are discouraging.

### 3.2. Biologics for Auto-Immune Conditions, Diseases Related to the Immune System, Genetic Disorders, and Other Types of Diseases Approved by the FDA in 2024

#### 3.2.1. Winrevair^TM^ (Sotatercept)

Sotatercept is a recombinant fusion protein indicated for the treatment of adult patients with pulmonary arterial hypertension (subclassified as PAH, WHO group 1) to increase exercise capacity, improve function, and delay disease progression [80].

PAH is a relatively rare disorder that is difficult to fully understand due to its complexity. It is associated with impaired quality of life and increased mortality. Patients with PAH are subclassified by the World Health Organization (WHO) into five groups on the basis of etiology. Those in Group 1 PAH are hemodynamically defined by a mean pulmonary artery pressure (mPAP) above 20 mmHg at rest, a pulmonary arterial wedge pressure (PAWP) of about 15 mmHg or less, and pulmonary vascular resistance (PVR) more than 2 Wood units by right heart catheterization. PAH can be caused by drugs or toxins, inherited genetic factors, or may develop in association with other medical conditions [81].

Sotatercept was first approved in March 2024 [82]. In patients with WHO Group 1 PAH and functional class II-III symptoms, adding sotatercept to background therapy improved 6 min walk distance in Phase II-III clinical trials. Pooled analysis from PULSAR (Phase II) and STELLAR (Phase III) showed improvements in pulmonary vascular resistance and NT-proBNP. However, according to the FDA, the first trial compared WINREVAIR and placebo in terms of 6 min walk distance (6MWD) at week 24 [80].

#### 3.2.2. Pisasky^TM^ (Crovalimab)

Paroxysmal nocturnal hemoglobinuria (PNH) is a serious, acquired, life-threatening disease characterized by hematologic events, such as hemolysis, thrombosis, and bone marrow dysfunction. These occur due to overactivation of the complement system—a part of the body’s immune response—highlighting the need for more effective and targeted treatments [17].

Crovalimab is a humanized complement C5 inhibitor indicated for the treatment of pediatric and adult patients from 13 years and older with PNH and a body weight of at least 40 kg. As a complement inhibitor, this drug increases a patient’s susceptibility to serious, life-threatening, or fatal infections caused by meningococcal bacteria (meningococcemia and/or meningitis) in any serogroup, including non-groupable strains. The treatment with crovalimab is contraindicated in patients with a serious previously unresolved Neisseria meningitidis infection due to immune system complications [83].

Some C5 inhibitors, such as eculizumab and ravulizumab, are recognized as the current standard of care for PNH treatment. However, crovalimab was approved for the first time in February 2024 for the treatment of this condition. It is a novel anti-C5 inhibitor administered every 4 weeks with a low-volume subcutaneous maintenance dose, allowing for potential self-administration. C5 inhibitors block (inhibit) C5, which plays a key role in the removal of red blood cells, thereby controlling PNH [84]. Importantly, inhibiting the complement system is not an inherently autoimmune-targeted mechanism of action, but it can be used to treat autoimmune-mediated conditions.

According to [85,86], crovalimab was evaluated in a pool of clinical trials that included more than 400 patients with PNH from more than 30 countries. Long-term follow-up results of the Phase I/II COMPOSER trial [86] showed that crovalimab was well accepted by patients with PNH. In the global, randomized, Phase III trial named COMMODORE 1 [87], treatment with crovalimab maintained disease control, was well tolerated, and preserved basal levels of fatigue, functioning, and PNH symptoms in patients previously treated with eculizumab. COMMODORE 2, another global, randomized, Phase III trial [88], demonstrated that crovalimab showed similar efficacy to eculizumab for endpoints of hemolysis control and transfusion avoidance. Crovalimab was also shown to have a safety profile consistent with that of other C5 inhibitors, and patients experienced rapid, sustained, and clinically meaningful improvements in symptoms such as fatigue and sustained improvements in functioning and PNH symptoms.

In a new single-arm study called COMMODORE 3 [89], crovalimab met its co-primary efficacy endpoints of hemolysis control and transfusion avoidance and was also shown to be well tolerated.

There are no studies currently underway addressing this drug, and although some results have been published, further evaluations over longer periods are needed to ensure the efficacy and widespread use of this medication in both children and adults.

#### 3.2.3. Nemluvio^TM^ (Nemolizumab)

Prurigo nodularis (PN) is a skin condition characterized by multiple firm nodules, ranging in color from flesh to pink that often appear on the outer parts of the arms and legs. The itching can be intense and persistent, and the condition can affect individuals of any age. PN often appears in people who also have other itchy skin conditions, like atopic dermatitis or long-standing chronic pruritus. The precise cause of PN is still not well understood, but it seems to stem from neurogenic inflammation in the skin, driven by various neuropeptides (substance P, calcitonin gene-related peptide, and vanilloid receptor subtype 1). Furthermore, patients with PN tend to have elevated levels of interleukin 31 (IL-31), a cytokine released by T cells that is strongly linked to itch sensation [90].

Nemolizumab was approved by the FDA on 12 August 2024 based on evidence from two clinical trials (OLYMPIA 1 and OLYMPIA 2) [91]. This drug is a humanized mAb and IL-31 receptor antagonist, blocking the signaling pathway responsible for itching. In conditions like atopic dermatitis and PN, nemolizumab also helps reduce inflammation and skin lesion severity by restoring the skin’s barrier function and supporting epithelial repair [92].

In OLYMPIA 1, a multicenter, placebo-controlled, Phase III randomized clinical trial [93], patients received nemolizumab monotherapy via subcutaneous injections of 30 mg or 60 mg, or placebo, administered every 4 weeks for 24 weeks. Results showed that a greater proportion of individuals treated with the drug reported a reduction in itching compared to those given the placebo [94].

In a double-blinded, multicenter, randomized trial [93], similar to OLYMPIA 1, adult patients with moderate-to-severe PN received an initial dose of 60 mg of nemolizumab, followed by subcutaneous injections of either 30 mg or 60 mg every four weeks for a total of 16 weeks, with the dose adjusted based on baseline body weight. A matching placebo was administered to the control group. At 16 weeks, the results showed that nemolizumab was effective, with more patients treated with the drug reporting improvement in itching compared to those in the placebo group. It was concluded that monotherapy with nemolizumab resulted in a significant reduction in both the signs and symptoms of PN [95].

Nemolizumab is the most recent drug approved by the FDA for PN. More biologics for this condition are in clinical trials (e.g., vixarelimab/KPL-716 and barzolvolimab/CDX-0159), which may yield new approvals [96].

#### 3.2.4. Niktimvo^TM^ (Axalitimab)

According to the FDA, axalitimab is a humanized mAb used to treat children and adults with chronic graft-versus-host disease (GVHD), a complication that can occur after receiving a bone marrow or stem cell transplant from a donor. It is indicated for patients who weigh at least 40 kg, have received at least two prior treatments (systemic therapy), and require additional treatment [97]. Axalitimab was approved in August 2024 as Niktimvo^TM^ [98].

NCT03604692 was a Phase I/Phase II open-label study [99] aimed to evaluate the safety, tolerability, and efficacy of axatilimab in children 6 or over with active cGVHD after at least two prior systemic therapy lines. In the Phase I trial, involving 17 patients, 3 mg/kg was defined as the optimal biologic dose and was administered once every 4 weeks. Two dose-limiting toxicities occurred with the 3 mg/kg dose given once every 2 weeks. In the Phase II cohort, involving 23 patients, the primary efficacy endpoint occurred with an ORR of 50% on the first day of cycle 7. Furthermore, in the first six cycles, the ORR was about 82%, an endpoint that supports regulatory approval. Responses were seen in all affected organs regardless of previous therapy. Fifty-eight percent of patients reported a significant improvement in cGVHD-related symptoms. No cytomegalovirus reactivations occurred. The study concluded that axatilimab is a promising novel therapeutic strategy with a favorable safety profile for refractory cGVHD [100].

#### 3.2.5. Ebglyss^TM^ (Lebrikizumab)

Atopic dermatitis is a common skin condition, affecting 2% to 10% of adults in developed countries. However, this chronic disease usually starts in infancy and causes dry skin, eczematous lesions, and lichenification. Its prevalence has risen two- to three-fold over the past few decades and may be related to decreased sun exposure and lower humidity levels [101].

The most recent approval by FDA for the treatment of adult and pediatric patients with moderate to severe atopic dermatitis was lebrikizumab (Ebglyss^TM^), which is a high-affinity IgG4 mAb that targets IL-13, blocking it from signaling through the IL-4Rα/IL-13Rα1 receptor complex, thereby reducing the release of inflammatory molecules such as cytokines, chemokines, and IgE [102].

To assess the effectiveness and safety of lebrikizumab, the trials ADvocate 1 [103], ADvocate 2 [104], and ADhere [105] were conducted in adolescent and adult patients with moderate-to-severe atopic dermatitis. On the basis of the results of those trials, it was concluded that lebrikizumab was effective in reducing the signs and symptoms of atopic dermatitis in adolescents, consistent with the overall results observed in the ADvocate and ADhere study population [106]. A Phase III, open-label study, namely ADcore [107], evaluated the efficacy and safety of the drug in a 52-week treatment. The results showed that a dose of 250 mg administered every two weeks was well tolerated and helped reduce atopic dermatitis symptoms while improving quality of life. Patients saw meaningful improvements by week 16, which continued to improve through to week 52 [108]

More biologic treatment options are available for atopic dermatitis, including tralokinumab-ldrm (approved by the FDA in 2021) and dupilumab. A systematic review and network meta-analysis (NMA) were carried out to compare the performance of lebrikizumab in the short term with that of other approved biologic treatments for moderate-to-severe atopic dermatitis in adults and adolescents. The studies of 22 monotherapies showed that by week 4, lebrikizumab had a greater capacity to reduce itch compared to tralokinumab, and it had a similar performance to dupilumab [109].

#### 3.2.6. Letybo^TM^ (LetibotulinumtoxinA)

Botulinum neurotoxin (BoNT) injections have become the most popular non-surgical cosmetic treatment, drawing more and more interest from cosmetic practitioners around the world [110]. When BoNT type A is injected into the skin, it is believed to have a lifting effect when used at a low concentration and variable dilution [111].

LetibotulinumtoxinA is a neuromuscular blocking agent that inhibits the release of acetylcholine. When injected into the muscle at therapeutic doses, letibotulinumtoxinA enters the nerve terminal and travels into the cytoplasm of the neuron. There, it breaks down SNAP25, a protein essential for acetylcholine release, leading to a dose-dependent decrease in muscle function. As the neurotoxin degrades and axonal sprouts form, muscle function begins to recover gradually. Over time, muscle reinnervation occurs, gradually reversing the pharmacological effects of letibotulinumtoxinA [112].

LetibotulinumtoxinA was approved based on evidence from three identical, randomized, double-blind, placebo-controlled Phase III trials (ClinicalTrials.gov Identifier: BLESS I [113]; BLESS II [114]; and BLESS III [115]). All the trials included adults between 18 and 75 years old who had moderate to severe glabellar lines at the maximum frown (score 2 or 3 on the Facial Wrinkle Scale (FWS)).

In the first trial (BLESS I), the subjects were randomized 3:1 to receive a treatment of letibotulinumtoxinA or placebo. Of those who received the treatment, 46.5% had a response compared to placebo (none of the placebo group responded to treatment) at week 4.

The second trial (BLESS II) was similar to BLESS I. At week 4, the results of this trial showed that 48.8% of the letibotulinumtoxinA group had temporary improvement in wrinkles compared to 1.9% of the placebo group.

In the BLESS III trial, a total of 355 subjects were randomized: 266 received treatment with letibotulinumtoxinA and 89 received a placebo. At week 4, 64.7% of those in the letibotulinumtoxinA group responded to treatment, while none of the placebo group responded. No deaths were reported during studies with letibotulinumtoxinA [115].

There are currently several botulinum toxins on the market, the most popular of which continues to be onabotulinumtoxinA (Botox^TM^). In Phase III clinical trials involving Chinese patients [116], letibotulinumtoxinA demonstrated non-inferiority to onabotulinumtoxinA [117]. However, there is a lack of data in the literature, and further studies in other countries are required to establish its efficacy compared to onabotulinumtoxinA.

#### 3.2.7. Hympavzi^TM^ (Marstacimab) and Alhemo^TM^ (Concizumab)

Hemophilia A and B are both rare genetic bleeding disorders linked to the X chromosome. They occur due to genetic mutations that disrupt the production of clotting proteins—specifically factor VIII in hemophilia A and factor IX in hemophilia B. Hemophilia A is more common, with an estimated rate of 1 case per 5000 live male births, whereas hemophilia B affects about 1 in 30,000 male births. Patients may bleed for a longer time than normal after an injury. When joint bleeding occurs repeatedly, it can cause long-term damage like severe joint disease (arthropathy), muscle loss, and even the formation of pseudo-tumors. These complications often lead to ongoing pain and difficulty moving the affected joints. Hemophilia A and B share similar clinical features, but there are some differences in bleeding frequency and factor replacement usage [118].

In 2024, the FDA approved two mAbs to prevent or reduce bleeding episodes related to hemophilia A and B. The first, marstacimab, which was approved on 11 October, is a human IgG1 mAb that targets tissue factor pathway inhibitor (TFPI). It is indicated for routine prophylaxis to help prevent or reduce the frequency of bleeding episodes in adolescents, adults, and elderly individuals with hemophilia A (without factor VIII inhibitors) or hemophilia B (without factor IX inhibitors) [22].

This IgG1 antibody attaches to a specific part of TFPI called Kunitz domain 2 (K2). By blocking TFPI, marstacimab enhances blood clotting. Normally, TFPI acts as a brake in the extrinsic pathway of coagulation by stopping key proteins like factor Xa and the tissue factor complex from producing thrombin [119].

The second approval was concizumab, a humanized IgG4 mAb and TFPI antagonist. It is administered subcutaneously and is designed to help manage bleeding in individuals with hemophilia A (congenital factor VIII deficiency) and FVIII inhibitors and in individuals with hemophilia B (congenital factor IX deficiency) and FIX inhibitors [27].

Concizumab binds to the K2 of TFPI, and by blocking it, this IgG4 antibody stops it from attaching to factor Xa (FXa) and prevents its action. This allows the FVIIa–tissue factor complex to produce enough FXa for proper clot formation. Due to its mechanism of action, concizumab is expected to be effective for both hemophilia A and B patients, regardless of the presence of inhibitors [120].

The mechanisms of action of marstacimab and concizumab are very similar, but the difference lies in the type of mAb. Marstacimab is an IgG1 antibody, while concizumab is an IgG4 antibody. Additionally, conzimumab is effective for both hemophilia A and B patients, with or without inhibitors, while marstacimab is effective only for hemophilia patients without inhibitors.

The efficacy of concizumab was evaluated in Explorer 7, a multicenter, open-label, Phase III trial [121], involving patients with hemophilia A or B, with inhibitors. The study compared the number of treated bleeding events between the treatment group and the no-prophylaxis group. Results showed that patients receiving concizumab prophylaxis experienced a lower annualized bleeding rate than those who did not receive prophylaxis [122].

Marstacimab was approved based on an open-label, multicenter study involving 116 adult and pediatric male patients with severe hemophilia A or B, without inhibitors. In the first six months, patients received either on-demand factor treatment or regular prophylactic factor treatment. After this phase, all patients switched to marstacimab prophylaxis for a year. Those initially treated on-demand saw their yearly bleeding rate drop from 3.8 to 3.2 with this drug, showing a clear improvement. Patients who started with prophylactic factor treatment had a bleeding rate of 7.85, which remained similar at 5.08 during treatment with marstacimab, thus indicating comparable results [123].

### 3.3. Biologic for Alzheimer’s Disease Approved by the FDA in 2024

#### Kisunla^TM^ (Donanemab)

Regarding the incidence and prevalence of AD, in 2015, around 46 million people were living with AD worldwide. This number is expected to reach over 74 million people by 2030. In 2018, about 50 million were diagnosed with AD worldwide, with the highest number of cases occurring in low-income and middle-income countries, as well as developed countries [124,125].

In 2023, around 55 million people were living with AD [126], and the number of patients affected by this condition is estimated to triple by 2050 worldwide [127,128].

There is some evidence suggesting that AD incidence and prevalence in some regions (e.g., the USA and Western Europe) might decrease over time due to lifestyle changes and higher investments in education and infrastructure. However, the reasons behind such a future trend remain unclear, as there is a lack of studies examining these patterns in other parts of the world [127].

Several studies have estimated the incidence of AD by state in the USA (e.g., Washington, Oregon, Idaho, Montana, Texas, California, Arizona, Wyoming, Oklahoma, and Alaska; these states carry the highest percentage of the estimated number of people that would be affected by AD between 2000 and 2025) [129]. However, there are few data for poorer regions [127]. Incorporating the aspect of social inequality more deeply into this research question could help to shed light on the impact of AD incidence and prevalence from country to country and region to region. Meanwhile, other fields of research, such as the pathogenesis of AD, are ongoing and not fully understood, with new hypotheses continually emerging.

Approved by the FDA in 2024, donanemab (an antibody that specifically targets the N-terminal pyroglutamate β-amyloid epitope, also referred to as LY3002813) is indicated for patients with mild cognitive impairment or with the mild dementia stage of the disease [128].

The FDA approval of this drug in 2024 was based on the study ID NCT04437511 addressing AD [130].

In TRAILBLAZER-ALZ (ClinicalTrials.gov Identifier: NCT03367403), a randomized Phase II clinical placebo-controlled trial within a blind period of up to 76 weeks and a 48-week follow-up period, involving patients aged 60 to 85 years old presenting intermediate tau proteins levels, early symptomatic AD, and elevated amyloid levels, showed that at week 24, donanemab had reduced amyloid plaques substantially [131].

This decrease in amyloid plaques highlights a significant finding from this study related to baseline amyloid levels and treatment outcomes with donanemab. In this regard, 40% of the subjects in the donanemab group achieved complete clearance of amyloid plaques within this 24-week period, with a measurement threshold of 24.1 CL (the unit of measurement for amyloid levels). However, the average baseline of those achieving complete amyloid clearance was substantially lower (92.8 CL on average) compared to those who had only partial clearance. These observations imply that participants who experienced partial amyloid clearance had higher average baseline amyloid levels (117.4 CL on average), which indicates that those starting the treatment with lower amyloid levels were more likely to achieve complete clearance within this 24-week period [131].

Table 5 shows data from the main clinical trials of authorized biologics for AD, along with the performance of donanemab relative to placebo.

Regarding all the immunotherapies approved for AD to date, donanemab demonstrated better efficacy than aducanumab in some of the main endpoints in clinical trials for AD drugs compared to placebo. Improvements in CDR-SB and ADAS-Cog 13 scores were also observed. Importantly, the measures used to analyze these outcomes differed between the biologics.

Aducanumab results are reported as change from baseline, representing a raw difference between baseline and week 78. Donanemab results are reported as adjusted mean change from baseline, which are derived from more specific statistical methods: MMRM (mixed-effect model repeated measures, which analyzes repeated measures overtime) analysis for CDR-SB and NCS2 (normalized clinical severity score 2, which analyzes clinical severity scores) analysis for ADAS-Cog 13. These methods provide a more refined estimate of effects, with greater control of the possible variability between patients and accounting for factors like baseline severity, thus yielding more reliable results.

Despite these methodological differences, donanemab results, such as adjusted mean changes from baseline of 1.72 in CDR-SB and 5.46 for ADAS-Cog 13 suggests a better slowing of disease progression and cognitive decline compared to aducanumab results 1.35 in CDR-SB and 3.76 for ADAS-Cog 13 (both biologics compared to placebo, where lower scores are better for these scales, indicating less impairment). Indicating donanemab may have better efficacy versus placebo. Moreover, aducanumab’s results may seem better because they are closer to 0 than donanemab’s results, but donanemab’s baseline scores are higher than aducanumab’s, indicating that donanemab’s study population had more severe cognitive and functional impairment at baseline. In addition, the statistical methodology used for donanemab’s results are more reliable, as previously discussed.

When analyzing donanemab and lecanemab, a significant slowing of disease progression was observed compared to the placebo, with the latter showing a slightly better outcome in terms of CDR-SB (a lower adjusted mean change), 1.21 vs. 1.72, respectively. Regarding the other primary endpoint analyzed, different scales were used for the two biologics in these studies (ADAS-Cog 13 and ADAS-Cog 14), thus hindering the comparison of results. However, both biologics achieved a substantial cognitive decline according to the results compared to the placebo.

For confirmation of these findings, specific clinical trials directly comparing these drugs with the same methodological approaches are required.

## 4. Conclusions

In summary, of the biologics approved in 2024, ten received ODD, except lebrikizumab. As seen in Table 3, this distinction considers whether the therapeutic indication was fully approved and included in the prescribing information or whether it received only ODD without subsequent approval for use, as explained in this paper. Nine biologics were designated first-in-class drugs, seven used the Fast Track route, seven received Breakthrough Therapy, and nine were granted Priority Review. Only three biologics used the Accelerated Approval program, and nine were first approved in the USA before receiving authorization in other countries. Importantly, in 2024, the drugs that gained the most from these programs and designations targeted cancer, with tarlatamab and zenocutuzumab utilizing nearly all of them, and zanidatamab being the only drug to benefit from every single designation and program [1].

In 2024, a high number of drugs were approved (50) for a range of diseases. Regarding biologics, 16 received authorization, and cancer continued to be the most targeted disease by biologics. Furthermore, no ADCs were approved in 2024, marking the second consecutive year without an approval in this drug class.

Of note, 2024 brought about the expansion of biologics for pediatric patients with the extension of indications for inotuzumab ozogamicin and dupilumab (both in 2017), as well as blinatumomab (2014). This is worth mentioning as in previous articles of this series, the authors highlighted the clear capacity of biologics to have their therapeutic indications extended to other diseases or populations.

In the context of neurodegenerative diseases, the FDA approved another biologic for AD, namely donanemab. As seen in this review, current evidence from clinical trials suggests that this biologic is more effective than previous ones in slowing AD progression, as demonstrated in clinical trials by improvements in scores such as CDR-SB and ADAS-Cog 13 compared to placebo. This work also highlights differences in the methods used to compare the biologics for AD approval to date with placebo. The method used for donanemab (NCS2 analyses and MMRM) provides more reliable estimates for considering baseline severity and other factors, as explained previously. However, while lecanemab gives slightly better CDR-SB outcomes than donanemab, direct comparison remains challenging due to differing assessment scales (ADAS-Cog13 vs. ADAS-Cog14). The study population used for the donanemab trial had greater baseline impairment, further supporting its clinical relevance. However, trials directly comparing these biologics using standardized methodologies are needed to definitively rank these immunotherapies and provide a more accurate assessment of their efficacy. Until then, data rank donanemab as a promising and superior option in efficacy than other drugs.

Although cancer was still the most targeted disease in 2024, following the trend in previous years, there was a strong indication of a widening in targeting diseases. In this regard, three mAbs were authorized for skin-related disorders, namely nemolizumab and cosibelimab, both IgG1 mAbs, and lebrikizumab, an IgG4 mAb. Notably, this is the year with the least number of autoimmune drug approvals compared to previous years. Further research into autoimmune conditions is needed to establish their onset and underlying cause.

Regarding the outcomes in clinical trials, all the biologics approved in 2024 were demonstrated to be superior or non-inferior to placebo or standard of care in the clinical trials reviewed herein.

A high number of first-in-class biologics received the green light in 2024, which highlights the pursuit of new approaches to tackle mechanisms of action on the part of pharmaceutical companies. Three bispecific mAbs were approved. This is significant given their enhanced efficacy to tackle two different targets, thus overcoming resistance to current single-target therapies. Furthermore, once again, a high number of biologics received the designation and approval of ODD. This reflects the channeling of continuous efforts into rare diseases. Of note, several clinical trials for some of the biologics mentioned herein, including lebrikizumab, are currently ongoing to explore their potential to treat other diseases, with the goal of securing the ODD designation from the FDA.

The pharmaceutical industry continues to demonstrate its effectiveness in addressing a wide range of diseases, accelerating innovation, and meeting unmet medical needs. Recent investments made by pharmaceutical companies, along with governments, are essential to drive future breakthroughs.

## Figures and Tables

**Table 1:** Correlation of total drug approvals vs. biologics approvals by the FDA.

Year	Total Drugs Approved (Biologics and NCEs)	Biologics Approved	References
2024	50	16 (32%)	[2]
2023	55	17 (31%)	[3]
2022	37	15 (41%)	[4]
2021	50	14 (28%)	[5]
2020	53	15 (28%)	[6]
2019	48	12 (25%)	[7]
2018	59	17 (29%)	[8]
2017	46	13 (28%)	[9]
2016	22	7 (32%)	[10]
2015	45	13 (29%)	[11]
2015–2024	431	139 (32%)	

This table includes publicly available data from FDA databases. Abbreviation: NCE—New Chemical Entity.

**Table 2:** Biologics approved by the FDA in 2024.

Drug Name	Class	Mechanism of Action	Original Approval Date	Pharmaceutical Company	Therapeutic Indication
Letybo ^TM^ (letibotulinumtoxinA-wlbg) [12]	Protein	Blocks cholinergic transmission at the neuromuscular junction by inhibiting the release of acetylcholine	29 February 2024	Hugel, Inc., Newport Beach, CA, USA	Temporary improvement in the appearance of moderate to severe glabellar lines associated with corrugator and/or procerus muscle activity in adult patients
Tevimbra ^TM^ * (tislelizumab-jsgr) [13]	Humanized IgG4	Inhibits T cell proliferation and cytokine production by binding to PD-1, blocking its interaction with PD-L1 and PD-L2	13 March 2024	BeiGen, Inc., Cambridge, MA, USA	Unresectable or metastatic ESCC and HER2 -(negative) gastric or gastroesophageal junction adenocarcinoma
Winrevair ^TM^ * (sotatercept-csrk) [14]	Recombinant fusion protein (recombinant ActRIIA-Fc)	Improves the balance between the proliferative (ActRIIA/Smad2/3-mediated) and anti-proliferative (BMPRII/Smad1/5/8-mediated) signaling to modulate vascular proliferation	26 March 2024	Merck Sharp & Dohme LLC, Rahway, NJ, USA	Pulmonary arterial hypertension
Anktiva ^TM^ (nogapendekin alfa inbakicept-pmln) [15]	Recombinant protein (IL-15 receptor agonist)	Stimulates the proliferation and activation of NK, CD8+ (positive), and memory T cells by binding to its receptor	22 April 2024	Altor BioScience, LLC, Miramar, FL, USA, an indirect wholly-owned subsidiary of ImmunityBio, Inc., Culver City, CA, USA	Unresponsive NMIBC with carcinoma in situ (CIS) with or without papillary tumors
Imdelltra ^TM^ * (tarlatamab-dlle) [16]	Bispecific humanized mAb	Promotes T cell activation, releases inflammatory cytokines, and lysis of DLL3-expressing cells by binding to DLL3 and CD3 expressed on cells	16 May 2024	Amgen Inc., Thousand Oaks, CA, USA	ES-SCLC with disease progression on or after platinum-based chemotherapy
Piasky ^TM^ * (crovalimab-akkz) [17]	Humanized IgG1	Binds with high affinity to the complement protein C5, inhibiting its cleavage into C5a and C5b, preventing the formation of the membrane attack complex	20 June 2024	Genentech, Inc., South San Francisco, CA, USA	Paroxysmal nocturnal hemoglobinuria
Kisunla ^TM^ (donanemab-azbt) [18]	Humanized IgG1	Acts against the insoluble N-truncated pyroglutamate amyloid-β peptide at position 3 (pGlu3-Aβ, AβpE3), reducing β plaques in the brain	2 July 2024	Eli Lilly and Company, Indianapolis, IN, USA	Alzheimer’s disease patients with mild cognitive impairment or mild dementia stage of the disease
Nemluvio ^TM^ (nemolizumab-ilto) [19]	Humanized IgG2	Inhibits IL-31 signaling by binding selectively to IL-31 RA	12 August 2024	Galderma Laboratories, L.P., Fort Worth, TX, USA	Prurigo Nodularis
Niktimvo ^TM^ * (axatilimab-csfr) [20]	Humanized IgG4	Binds to CSF-1R expressed on monocytes and macrophages	14 August 2024	Incyte Corporation, Wilmington, DE, USA	Chronic Graft-versus-Host Disease
Ebglyss ^TM^ (lebrikizumab-lbkz) [21]	Humanized IgG4	Binds with high affinity and slow off-rate to IL-13 and allows IL-13 to bind to IL-13Rα1 but inhibits human IL-13 signaling	13 September 2024	Eli Lilly and Company, Indianapolis, IN, USA	Moderate-to-severe atopic dermatitis
Hympavzi ^TM^ * (marstacimab-hncq) [2,22]	Human IgG1	Binding and inhibiting the factor Xa activity through the binding of TFPI to the factor Xa	11 October 2024	Pfizer Inc., New York, NY, USA	Prophylaxis to prevent bleeding episodes related to hemophilia A and B
Vyloy ^TM^ * (zolbetuximab-clzb) [23]	Chimeric (mouse/human) antibody, with regions derived from human IgG1	Depletes CLDN18.2-+(positive) cells via antibody-dependent cellular cytotoxicity (ADCC) and complement-dependent cytotoxicity (CDC)	18 October 2024	Astellas Pharma US, Inc., Northbrook, IL, USA	First-line treatment of adult patients with locally advanced unresectable or metastatic HER2-negative gastric or gastroesophageal junction adenocarcinoma whose tumors express claudin
Ziihera ^TM^ * (zanidatamab-hrii) [24]	Bispecific humanized IgG1	Induces CDC, ADCC, and ADCP by binding to two extracellular sites on HER2	20 November 2024	Jazz Pharmaceuticals Ireland Limited, Dublin, Ireland	Unresectable or metastatic HER2+ (IHC 3+) biliary tract cancer
Bizengri ^TM^ * (zenocutuzumab-zbco) [25]	Bispecific humanized IgG1	Inhibits HER2:HER3 dimerization and prevents NRG1 binding to HER3, also mediates ADCC by binding to the extracellular domains of HER2 and HER3	4 December 2024	Merus NV, Utrecht, The Netherlands	Advanced, unresectable, or metastatic NSCLC and pancreatic adenocarcinoma
Unloxcyt ^TM^ (cosibelimab-ipdl) [26]	Human mAb IgG1	Binds to PD-L1 and blocks the interaction between PD-L1 and its receptors PD-1 and B7.1	13 December 2024	Checkpoint Therapeutics, Inc., Waltham, MA, USA	Metastatic mCSCC or laCSCC
Alhemo ^TM^ * (concizumab-mtci) [27]	Humanized IgG4	Enhances factor Xa production during the initiation phase of coagulation, leading to improved thrombin generation and clot formation	20 December 2024	Novo Nordisk Inc., Bagsværd, Denmark	Routine prophylaxis to prevent bleeding episodes in hemophilia A and B

This table includes publicly available data from FDA databases; * Orphan Drug Designation granted by the FDA; Abbreviation: ESCC—esophageal squamous cell carcinoma; HER2—human epidermal growth factor receptor 2; PD-1—programmed death 1; PD-L1 and PD-L2—programmed death ligands 1 and 2; NMIBC—non-muscle invasive bladder cancer; ES-SCLC—Extensive stage small cell lung cancer; DLL3—Delta-like ligand 3; IHC—immunohistochemistry 3; CLDN18.2—Cloudin protein 18 isoform 2; CDC—complement-dependent cytotoxicity; ADCC—antibody-dependent cellular cytotoxicity; ADCP—antibody-dependent cellular phagocytosis; NSCLC—non-small cell lung cancer; mCSCC—metastatic cutaneous squamous cell carcinoma; laCSCC—locally advanced CSCC; ActRIIA-Fc—activin receptor type IIA-Fc; BMPRII—bone morphogenetic Protein Receptor Type II; CSF-1R—colony-stimulating factor-1 receptors; IL—interleukin; and TFPI—tissue factor pathway inhibitor; CD—cluster of differentiation; NK—natural killer.

**Table 3:** Orphan Drug Designations granted by the FDA from 2015 to 2024.

Year	Biologics Approved	Orphan Drug Designations Granted for New Biologics
2024	16	10 (63%)
2023	17	9 (53%)
2022	15	7 (53%)
2021	14	7 (50%)
2020	15	10 (66%)
2019	12	9 (75%)
2018	17	13 (76%)
2017	13	5 (38%)
2016	7	2 (28%)
2015	13	7 (53%)
2015–2024	139	70 (50%)

This table includes publicly available data from FDA databases, such as the Search Orphan Drug Designations and Approvals database of the USA Department of Health and Human Services.

**Table 4:** Orphan Drug Designation granted for biologics in 2024.

I Hematologic and Immunologic Indications		
Biologic	Designation	
Concizumab	ODD approved: Hemophilia A and B	
Marstacimab	ODD approved: Hemophilia A/B (with/without inhibitors)	
Crovalimab	ODD approved: Paroxysmal nocturnal hemoglobinuria	
Axatilimab	ODD approved: Chronic graft-versus-host disease	
II Oncologic indication		
Biologic	Designation	Regulatory status
Zenocutuzumab	ODD approved: Pancreatic cancer	—
Zanidatamab	ODD approved: Biliary tract cancer (accelerated approval)	ODD not approved for gastric cancer, including cancer of the gastroesophageal junction
Zolbetuximab	ODD approved: Gastric cancer	—
Tarlatamab	ODD: Small cell lung cancer	—
Tislelizumab	ODD approved: Esophageal cancer, gastric/gastroesophageal junction cancer	ODD not approved for hepatocellular and nasopharyngeal carcinoma
III Pulmonary		
Biologic	Designation	Regulatory Status
Sotatercept	ODD approved: Pulmonary arterial hypertension	Revoked or withdrawn: Beta-thalassemia intermedia/major, MDS-associated anemias
Lebrikizumab	—	ODD not approved for idiopathic pulmonary fibrosis

This table includes publicly available data from the Search Orphan Drug Designations and Approvals database of the USA Department of Health and Human Services. Abbreviation: ODD—Orphan Drug Designation; MDS—Myelodysplastic Syndrome.

**Table 5:** Clinical Trial Outcomes: aducanumab, lecanemab, and donanemab vs. placebo.

Biologics for AD	Clinical Outcome	Time Point	Mean Baseline	Change from Baseline	Adjusted Mean Change from Baseline	Study
Aducanumab	CDR-SB	Week 78	2.51	1.35	-	NCT02484547 (Phase III)
	ADAS-Cog 13		22.246	3.763		
Placebo	CDR-SB		2.47	1.74		
	ADAS-Cog 13		21.867	5.162		
Lecanemab	CDR-SB	Week 72	3.17	-	1.21	NCT03887455 (Phase III)
	ADAS-Cog 14		24.45		4.140	
Placebo	CDR-SB		3.22		1.66	
	ADAS-Cog 14		24.37		5.581	
Donanemab	CDR-SB	Week 76	3.92	-	1.72	NCT04437511 (Phase III)
	ADAS-Cog 13		28.53		5.46	
Placebo	CDR-SB		3.89		2.42	
	ADAS-Cog 13		29.16		6.79	

Abbreviation: AD—Alzheimer’s disease; CDR-SB—Clinical Dementia Rating Scale–Sum of Boxes; ADAS-Cog 13—Alzheimer’s Disease Assessment Scale–13-item Cognitive Subscale; ADAS-Cog 14—Alzheimer’s Disease Assessment Scale–14-item Cognitive Subscale.

## Data Availability

U.S. Food and Drug Administration: Search Orphan Drug Designations and Approvals: https://www.accessdata.fda.gov/scripts/opdlisting/oopd/index.cfm (accessed on 26 June 2024); FDA Purple Book: https://purplebooksearch.fda.gov/results?query=nirsevimab&title=Beyfortus (accessed on 26 June 2024); FDA Orange Book: https://www.fda.gov/drugs/drug-approvals-and-databases/orange-book-data-files (accessed on 26 June 2024); U.S. Food and Drug Administration: Designating an Orphan Product: Drugs and Biological Products: https://www.fda.gov/industry/medical-products-rare-diseases-and-conditions/designating-orphan-product-drugs-and-biological-products (accessed on 26 June 2024).
